# Native Japanese Polygonaceae Species as Potential Native Insectary Plants in Conserving Indigenous Natural Enemies

**DOI:** 10.3390/insects16020232

**Published:** 2025-02-19

**Authors:** David Wari, Junichiro Abe, Toshio Kitamura

**Affiliations:** 1 Western Region Agricultural Research Center (WARC), National Agriculture and Food Research Organization (NARO), Fukuyama 721-8514, Hiroshima, Japan; 2 Institute of Plant Protection (NIPP), National Agriculture and Food Research Organization (NARO), Tsukuba 305-8666, Ibaraki, Japan

**Keywords:** conservation biological control (CBC), natural enemies, invasive plants, organic farming, *Orius* spp., *Persicaria lapathifolia*, *Persicaria longiseta*

## Abstract

Conservation biological control (CBC) is the application of agricultural practices that utilize insectary plants to conserve and enhance natural enemies, thereby increasing their efficiency to suppress pests. Most of the insectary plants used in CBC are non-native invasive insectary plants, which are costly and pose threats to the local ecosystems and biodiversity. Alternative to non-native insectary plants, the use of native plants is proposed. Hence, the aim of this study is to identify native plant species that can be used as alternatives to non-native insectary plants to conserve and promote indigenous natural enemies (INEs) for sustainable pest management. To achieve this, first, we bio-prospected the surrounding habitats of organic fields in the western region of Japan (i.e., Hiroshima Prefecture) to identify native plant species as prospective native insectary plants. As a result, among various Japanese native plants surveyed, Polygonaceae plant species seem to host a variety of INEs, showing potential as a native insectary plant. We then conducted open field experiments to test the role of Polygonaceae plants in promoting INEs, thereby indirectly suppressing pest densities on vegetable crops such as eggplants. Results show that significantly high densities of INEs (green lacewing, *p* = 0.024; *Orius* spp., *p* = 0.001: GLM) were observed on eggplants with Polygonaceae plants compared to eggplants without Polygonaceae plants, leading to a significant reduction in pest densities (thrips, *p* = 0.000; whiteflies, *p* = 0.002: GLM) on the eggplants with Polygonaceae plants. Furthermore, molecular analysis revealed that *Orius* spp., as a representative INE in this study, migrated from Polygonaceae plants to eggplants, suggesting that Polygonaceae plants may conserve and promote INEs to vegetable crops, resulting in pest suppression. Here, we discuss the roles of Polygonaceae plants (and other native plants) in regulating pest densities on crops.

## Simple Summary

Manipulating a natural vegetation to regulate pest densities is a crucial strategy within conservation biological control (CBC). Much of the CBC has revolved around the non-native invasive species that, in many ways, are causing concerns among local farmers, hence the need for alternate but noninvasive insectary plants. In this study, we bio-prospected potential native plants that conserve indigenous natural enemies and their potential role in regulating pests on vegetable crops. Field surveys revealed Japanese native Polygonaceae species as native plants of interest. Interplanting Polygonaceae plant species with eggplants resulted in reduced pest populations compared to eggplant without Polygonaceae plants. Reduced pest numbers could be related to an increased number of natural enemies, possibly supported by the native Polygonaceae plants. Combined with other results, preliminary results show that Polygonaceae species may serve as a native insectary plant species in supporting indigenous natural enemies.

## 1. Introduction

To manage crop damage and losses by insect pests, farmers mostly rely on pesticides. However, excessive use of pesticides induces pest resistance and, with climate change at the helm, evolutionary changes could potentially trigger the movement of destructive pest species globally threatening the current pest control efforts in place [1]. To minimize the usage of pesticides and its negative impact on the environment, organic farming has been suggested and promoted by the government and research organizations. Organic farming is a type of agriculture system that uses agricultural farming techniques that causes minimal to no environmental impact on the local ecosystems and biodiversity [2]. One of the farming techniques adopted to control pests in organic farming is biological control. A biological control method used in organic farming that is not new but trending of late is the engineering of agroecosystems. The classical term herein is conservation biological control (hereafter referred to as CBC), which involves engineering of habitats with the aim of promoting natural enemies to regulate pest occurrences [3].

By and large, mostly non-native horticultural insectary plants have been used throughout CBC as hosts for natural enemies. While non-native horticultural insectary plants are well recognized and documented to enhance biological pest control, non-native insectary plants are somewhat causing concerns among locals via threats to native ecosystems and biodiversity. Nonetheless, excessive use of non-native insectary plants can be minimized by utilizing native plants. Essentially, native plant species are critical in maintaining biodiversity and a healthy ecosystem. From the agronomic and conservation perspective, native plants can be beneficial in many ways; they are adapted to the local environmental conditions [4], require little maintenance costs [5], have no risk of becoming invasive [4], and are readily available to natural enemies, providing food resources, shelter, and breeding grounds [6]. Unlike non-native insectary plants, research on utilizing native plants to conserve and promote natural enemies in CBC for pest control is still very patchy.

In Japan, the following flowering plants have showed the potential to harbor and/or promote natural enemies: white clover (*Trifolium repens* L. (Fabaceae)) and *Gramineae* plants [7], *Rudbeckia hirta* L. (Asteraceae) [8], *Salvia farinacea* Benth. (Lamiaceae) [9], *Verbena × hybrida* Voss. (Verbenaceae) [10], *Scaevola aemula* R.Br. (Goodeniaceae) [11], *Cleome hassleriana* Chod. (Cleomaceae), *Sesamum indicum* L. (Pedaliaceae) [12], and *Setaria viridis* Beauv (Poaceae) [13]. However, most, if not all, of these plants and/or grasses reported so far are non-native horticultural insectary plants. Little is known or reported on the use of native plants as ecologically sound alternatives to supporting indigenous natural enemies (hereafter referred to as INEs). In this study, we surveyed the agroecological habitats surrounding organic fields to identify native plant species that have the potential to conserves INEs. We identified native plant species as potential native insectary plants and tested their role in conserving and promoting INEs that can regulate pest occurrences on vegetable crops. Our preliminary data show that native plants have the potential to conserve and promote INEs. Here, we discuss the role of native plants as better alternative to non-native invasive insectary plant species in supporting diversity of INEs and their potential role in delivery of function in terms of pest suppression. Furthermore, from these preliminary results, we propose further surveys and experiments to build towards advancing the concept of sustainable CBC in organic farming in Japan through native insectary plants.

## 2. Materials and Methods

### 2.1. Study Site

The native plants surveys were conducted in organic fields in Jinsekikogen-town (34°51′14″, 133°12′59″), Higashi-Hiroshima (34°29′12″, 132°39′6″), and the National Agriculture and Food Research Organization (NARO), Western Region Agricultural Research Center (WARC) (NARO-WARC) research field (34°30′10″, 133°23′18″) in Fukuyama City, Hiroshima Prefecture, (Western) Japan. For the organic fields in Jinsekikogen-town and the Higashi-Hiroshima, the vegetation is almost surrounded by bushes and shrubs, with no applications of any synthesized chemical compounds such as pesticides, herbicides, and fertilizers. On the other hand, while no synthesized chemical compounds were used in NARO-WARC fields, since the fields are used in rotation between research groups, pesticides have been used to control other pests and diseases.

### 2.2. Surveying of Native Japanese Plants

Native plants inhabiting the field margins, hedgerows, and weedy areas surrounding the organic farms in Western Japan were surveyed from July to October between the years 2023 and 2024. The surveying months were selected to coincide with the peak cultivation periods of agriculturally important vegetables such as eggplants (*Solanum melongena* L. Solanaceae) and spring onions (*Allium fistulosum* L. Amaryllidaceae) in Western Japan. Essentially, plants at their floral stages were identified and the floral parts tapped five–ten times onto a plastic tub (40 cm by 20 cm) and the number of arthropods that fell onto the white tub were quantified and recorded. Furthermore, the number of branches, leaves per branch, as well as flower per plant were recoded for shrubby plants, while, for creeping plants with a web of branches that could not be quantified, a 50 cm by 50 cm quadrat was used to measure the area and quantify the number of branches, number of leaves, and number of flowers to calculate the density of the plants and correlate it with the number of arthropod species. However, since a variety of native plants with different growth patterns were observed, the arthropod trends on native plants are presented as observed or absent for each sampling month. The detailed data can be produced upon request.

Similarly, Japanese native plant seeds (native plants that have been cultivated in controlled settings for preservation of the native flowering plants of Japan) that were identified from the 2023 field surveys were ordered from ESPEC Mic Corp. (Osaka, Japan), germinated, and transplanted onto the NARO-WARC research field to confirm if the native plants harbored INEs. Additionally, *Ocimum basilicum* L. (Lamiaceae), *O. tenuiflorum* L. (Lamiaceae), and *Trifolium pratense* L. (Fabaceae), non-native insectary plants known to conserve and promote natural enemies, were also geminated and planted in the fields to compare with the native plant species. Arthropod sampling was as discussed above. Moreover, *Orius* spp. specimens were also sampled and stored in a glass container filled with 99% EtOH for further experiments.

### 2.3. Semi-Field Studies on the Effect of Japanese Native Polygonaceae Plant Strips on Arthropod Species on Eggplants

**Figure A1:**
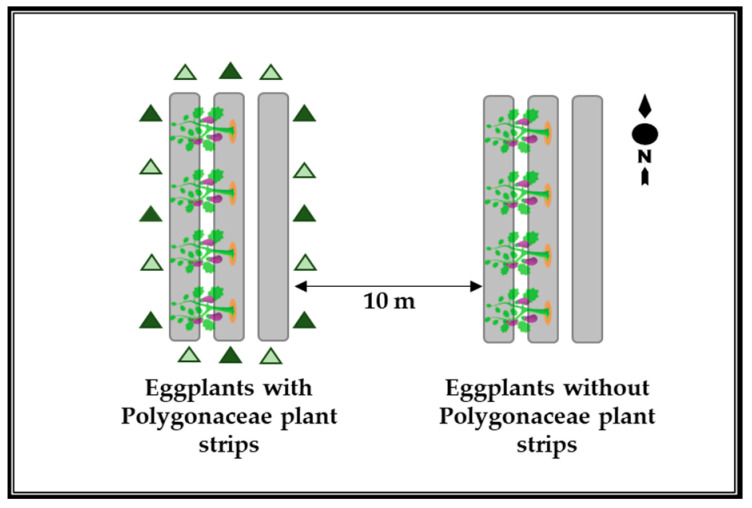
Schematic layout of the field experiments carried out in WARC-NARO experimental fields. Two treatments (eggplants with Polygonaceae plant strips and another without Polygonaceae plant strips) replicated three times. Three beds per treatment with a total of 24 plants (8 plants for each bed). Eggplants were spaced 1 m apart from each other on the beds. Each bed was 1 m by 10 m and the beds were spaced 1.5 m apart. Each treatment therefore covered a total crop area of 70 m^2^. For eggplant with Polygonaceae plant strips, an additional 1 m from the edge beds was used for planting Polygonaceae plantings, therefore covering a total crop+Polygonaceae area of roughly 100 m^2^. Treatments and replicates were separated by 10 m (a 100 m^2^ in terms of area) bare zone so that interaction between the treatments and replicates was minimized. Covering both the crop area and the non-crop area, a total of 1620 m^2^ of land area was used in this study. A randomized block design was used to assess the impact of native plant on regulating pest densities on eggplants. For the eggplants with Polygonaceae plant strips, *P. longiseta* and *P. lapathifolia* were interplanted around the eggplant beds as shown above with light green and dark green triangles, respectively.

To determine the role of Japanese native plants on conserving and promoting INEs resulting in suppression of pest (eggplant pests of agricultural importance such as aphids and thrips) populations, semi-field studies were performed at NARO-WARC research field. Japanese native Polygonaceae plant (*Persicaria longiseta* (Bruijn) Kitagawa 1937 and *P. lapathifolia* (L.) Delarbre 1800) strips were planted surrounding the eggplant beds (see Figure A1). Two treatments, eggplants with Polygonaceae plant strips and eggplants without Polygonaceae plant strips with three biological replicates each, were conducted. Each replicate had 24 eggplant plants. Since the field was limited to carry out the experiment, each treatment with their replicates were spaced 10 m to each other. After field ploughing, prior to bed preparations, S604 (NPK with 16:10:14 ratio (Zen-noh West Co., Ltd., Hiroshima, Japan)), a readily available fertilizer, was applied with rates according to the recommendations by the manufacturer. To mimic the eggplant cultivations in Western Japan, eggplant seeds were sowed on 8 March and transplanted on 21 May 2024. The eggplant cultivar used in this study was the medium in length eggplant cv. Kuromasari LF. Polygonaceae seeds were sowed in early April and transplanted when the plantlets were about 8 weeks old following the transplantation of eggplants on 29 May 2024. Arthropod surveys (both pests and INEs) were performed on at least a fortnight basis from 19 June through to 29 October. Eggplant seedlings were 15 weeks old (from sowing to the start (19 June) of the survey) when the survey was started. Six leaves, two each from the base of the plant, the mid-section, and the canopy of the eggplants, were surveyed to determine the population trends of arthropods. The six leaves were used as representative of the whole plant. Due to the smaller land area used in this study (see Figure A1), all 24 eggplants for each replicate per treatment were surveyed to clearly capture the population trends of pests and INEs on eggplants.

Native Polygonaceae plants were also surveyed to determine the population dynamics of pests and INEs. Since the whole plant was laborious to survey, one branch each with its flower buds for each plant for *P. longiseta* and *P. lapathifolia* were tapped five–ten times onto a plastic tub (40 cm by 20 cm) and the arthropods that fell onto the plastic tub were quantified. The arthropod numbers on the Polygonaceae plants are expressed as number of arthropods per branch per plant surveyed.

**Figure A2:**
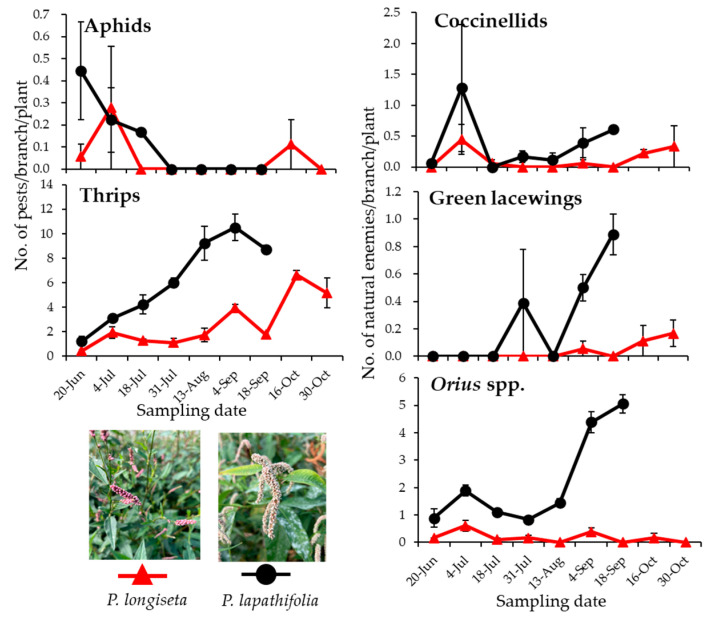
Population dynamics of pests (aphids and thrips) and INEs (coccinellid, green lacewings, and *Orius* spp.) on Polygonaceae plants *Persicaria longiseta* and *P. lapathifolia*. Red lines represent arthropod species surveyed on *P. longiseta* and black lines represent arthropod species surveyed on *P. lapathifolia* (n = 6, error bars: SE).

Pests (such as aphids, thrips, and whiteflies) and INEs (e.g., coccinellids and green lacewings) were all collectively surveyed at their family level. For instance, there are multiple species of aphids that use eggplants as hosts, such as *Aphis gossypii* [14], *Aulacorthum solani* [15], and *Macrosiphum euphorbiae* [16], to name a few. Furthermore, thrips species such as *Thrips palmi* and *Frankliniella intonsa* [13] are also reported to infest eggplants. To identify each aphid species in the field and, at the same time, identify other pests and natural enemies in the field is time-consuming and laborious. Therefore, pests such as aphids, thrips, and whiteflies were generally identified and quantified as aphid species, thrips species, and whitefly species, respectively. Likewise for coccinellids and green lacewing surveys. However, in the case of *Orius* species, since increased densities of *Orius* spp. were observed on Polygonaceae plants, species identification at the species level was essential. Therefore, *Orius* spp. Adults, as the INE of interest in this study from both eggplants and *P. lapathifolia*, were collected and stored separately in 99% EtOH for identification and species composition analysis, as well as detection of *P. lapathifolia* DNA using molecular tools. *Orius* spp. from *P. lapathifolia* were chosen because of their higher densities compared to *P. longiseta* (See Figure A2).

### 2.4. Orius *spp.* DNA Extraction

DNA from the adult *Orius* spp. specimens collected from eggplants and *P. lapathifolia* was extracted using 2× STE buffer (200 mM NaCl, 20 mM Tris-Cl, pH 8.0, and 2 mM EDTA) and Proteinase K (TaKaRa Bio Inc., Shiga, Japan). In brief, *Orius* spp. specimen (1 individual each) was homogenized in a sterile 1.5 mL tube. STE buffer and Proteinase K were then added, followed by heat treatment, once at 55 °C for 30 min followed by 95 °C for 15 min and a subsequent centrifugation at 20,000× *g* for 3 min. The resultant supernatant was then used as the DNA template for PCR.

### 2.5. Molecular Identification of Orius *spp.* Using Multiplex PCR

PCR was performed as described in Hinomoto et al. [17] with minor modifications. Essentially, 5× KAPA2G Robust HotStart ReadMix with Dye (KAPA Biosystems, Wilmington, MA, USA) was used as the polymerase with primer sets described in Hinomoto et al. [17]. Amplification was performed in a TP350 PCR Thermal Cycler Dice Touch (TaKaRa Bio Inc.) with the following conditions: 1 cycle at 94 °C for 3 min, followed by 30 cycles of 94 °C for 15 s, 56 °C for 15 s, and 72 °C for 1 min, with a subsequent final extension at 72 °C for 7 min. The PCR products were electrophoresed on a 1% agarose gel in TAE (40 mM Tris, 40 mM acetic acid, and 1 mM ethylenediaminetetraacetic acid) stained with SYBR Safe DNA Gel Strain (Invitrogen, San Francisco, CA, USA) and observed under WSE-5400-UP Printgraph Classic (ATTO Corporation, Tokyo, Japan) mounted on a Safe Imager Blue Light Transilluminator (Invitrogen, San Francisco, CA, USA). *Orius* spp. were estimated from the DNA bands on the 1% agarose gel as reported in Hinomoto et al. [17]. Ideally, *O. minutus* (L.) should show an approximate PCR fragment length of 910 bp, *O. sauteri* (L.) with 660 bp and 320 bp, *O. strigicollis* (Poppius) with 680 bp and 490 bp, and *O. nagaii* Yasunaga with 610 bp and 190 bp. *Orius tantillus* (Motchulxky, 1863) does not occur in the main island of Japan so *O. tantillus* amplification is not expected.

### 2.6. Detection of P. lapathifolia DNA in Adult Orius *spp.* Individuals

*Orius* spp. DNA samples used in identification and species composition were also used in the detection of *P. lapathifolia* DNA. PCR was performed using the 5× KAPA2G Robust HotStart ReadMix with Dye using the primer sets Pl_F 5′ CTTTCCAAGGCCCACCTCAC 3′ and Pl_R 5′ GAACATGATCCCCACCGGAC 3′. Amplification was performed in a TP350 PCR Thermal Cycler Dice Touch with the following conditions: 1 cycle at 95 °C for 3 min, followed by 40 cycles of 95 °C for 15 s, 55 °C for 15 s, and 72 °C for 30 s, with a subsequent final extension at 72 °C for 7 min. The PCR products were electrophoresed on a 1% agarose gel in TAE stained with SYBR safe and observed under WSE-5400-UP Printgraph Classic mounted on a Safe Imager Blue Light Transilluminator. *Persicaria lapathifolia* DNA was estimated from the DNA bands on the 1% agarose gel. Ideally, *P. lapathifolia* DNA in the gut of *Orius* spp. should show an approximate PCR fragment length of 500 bp.

### 2.7. Statistical Analysis

Statistical analyses were performed only for the arthropod pests (aphids, whiteflies, and thrips) and INEs (coccinellids, green lacewings, and *Orius* spp.) observed on eggplants. The pests and INE counts were tested for normality (Shapiro–Wilk test and Lilliefors test) and homogeneity (Bartlett’s Test) using open-source software OpenStat (https://smerser.github.io/OpenStat/OpenStatMain.htm accessed on 21 December 2024). The total number of pests and INEs were then transformed using ‘x + 1’ where necessary. The effects of ‘treatments’ (eggplants with Polygonaceae plant strips and without Polygonaceae plant strips) and ‘sampling interval’ (weeks) factors and their interaction on the densities of pests and natural enemies on eggplants were analyzed using Sum of Squares by Regression, a partial Generalized Linear Model (GLM) by OpenStat software ver. 30.0. Chi-square test analysis for detection of *P. longiseta* DNA in the gut of *Orius* spp. was performed using the Microsoft excel software.

## 3. Results

### 3.1. Japanese Native Plants Survey

In the search for native plants that conserve and promote INEs, we surveyed the agroecosystems surrounding the organic farms in Western Japan. A two-year (2022–2023) survey revealed more than 20 flowering wild plant species (both native and non-native plant species) (Table 1). The collected flowering plants were cross-referenced with the wild plants listed in [18,19] to confirm that the plants were of the correct species. While most of the flowering plants surveyed showed frequent occurrences of known pests such as aphids, thrips, and spider mites, several native plants seem to harbor INEs. Native plants from the Polygonaceae family such as *P. filiformis* (Thunb.) Nakai, *P. lapathifolia*, *P. longiseta*, *P. orientalis* (L.) Spach (a non-native but neutralized ornamental flowering plant in Japan), and *P. pubescens* (Blume) H.Hara seem to harbor INEs such as *Orius* spp., coccinellids, green lacewings, phytoseiid mites, hoverfly species from the Syrphidae family, and the occasional occurrence of parasitoids, which are important natural enemies of agriculturally important pests such as aphids, thrips, and spider mites. Other native plants such as *Commelina communis* (L.) (Commelinaceae), *Eclipta prostrata* (L.) L. (Asteraceae), *Eupatorium japonicum* Thunberg ex Murray. (Asteraceae), *Impatiens textori* Miq. (Balsaminaceae)*, Paederia foetida* L. (Rubiaceae), *Patrinia scabiosifolia* Link (Caprifoliaceae), and *Salvia japonica* Thund (Lamiaceae) were also some of the native plants observed to harbor INEs and could be considered as potential candidates in future studies (Table 1).

**Figure 1:**
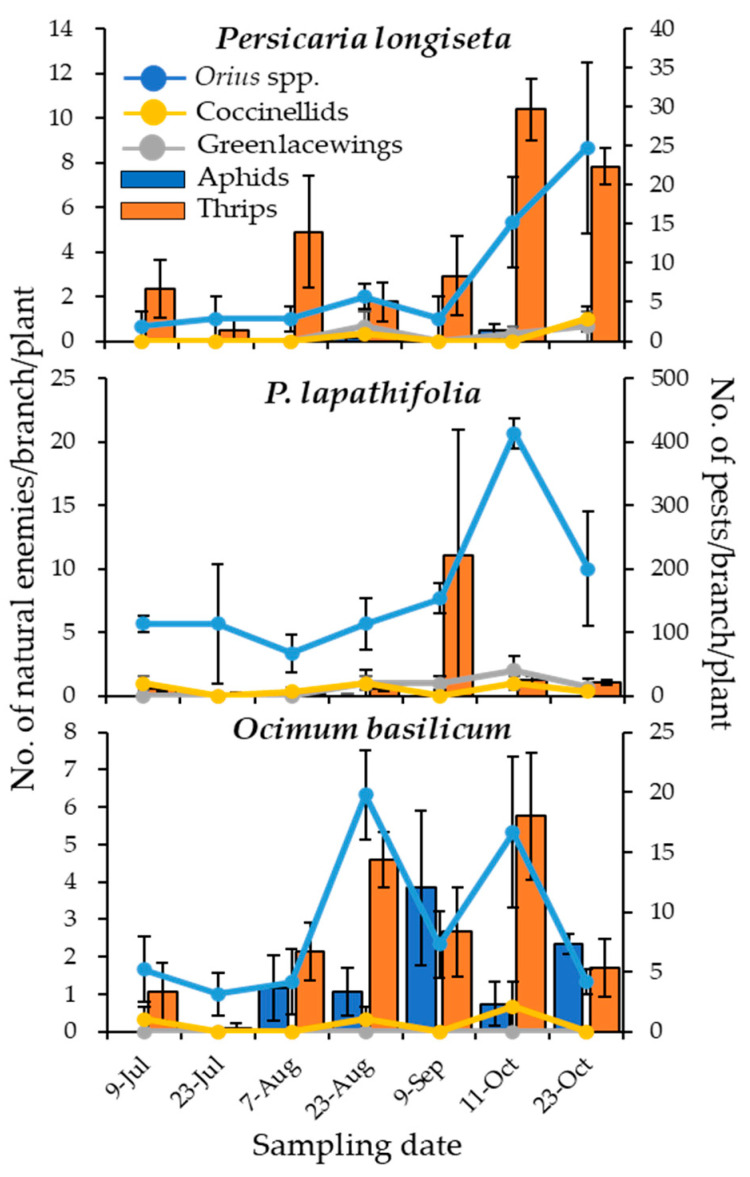
Population dynamics of pests (blue bar graphs for aphids and orange bars for thrips) and INEs (blue lines for *Orius* spp., orange lines for coccinellid beetles, and grey lines for green lacewings) on *Persicaria longiseta*, *P. lapathifolia*, and *Ocimum basilicum*. (n = 3, error bars: SE.).

*Persicaria longiseta* and *P. lapathifolia* showed a relatively increased number of INEs, therefore, both plants were selected as representatives of the Polygonaceae species and the population trends of pests (aphids and thrips) and INEs (*Orius* spp., coccinellids, and green lacewings) were compared to a known non-native insectary plant such as *O. basilicum*. Results show that thrips were mostly dominant on the *P. longiseta* and *P. lapathifolia*, with few to no occurrences of aphids, while both thrips and aphids were observed to occur almost simultaneously throughout the survey on *O. basilicum* (Figure 1). As for the INEs, while sporadic but low numbers of coccinellids and green lacewing occurrences were observed on *P. longiseta*, *P. lapathifolia*, and *O. basilicum*, a large number of *Orius* spp. was observed on the three plants but substantially high on Polygonaceae plants, especially *P. lapathifolia*. As is a known phenomenon, the population trends of *Orius* spp. somewhat followed that of the thrips on Polygonaceae plants, suggesting that Polygonaceae plants could be an important native host plant of *Orius* spp. with thrips as the food source. From these results, we selected *P. longiseta* and *P. lapathifolia* as potential native insectary plants that can conserve and promote *Orius* spp. and possibly other INEs.

### 3.2. Preliminary Field Studies on the Potential Role of Polygonaceae Plants as Insectary Plants

**Figure 2:**
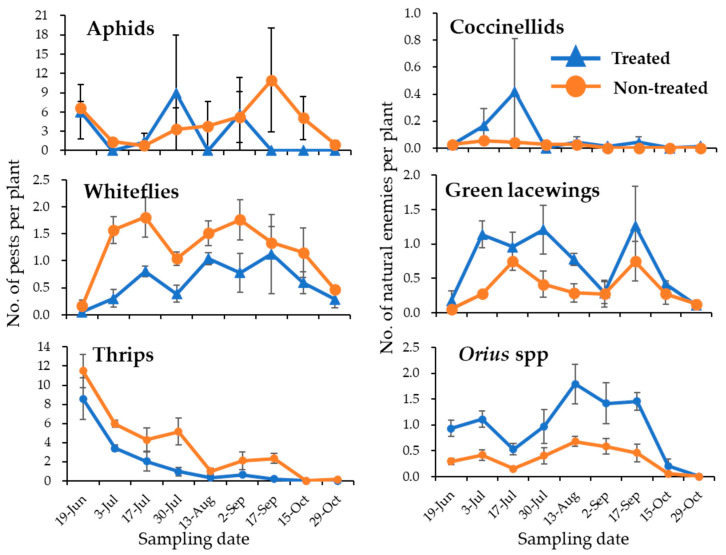
Population dynamics of pests (aphids, whiteflies, and thrips) and INEs (coccinellid, green lacewings, and *Orius* spp.) on eggplants. Blue lines (treated) represent eggplants with Polygonaceae plant strips and orange lines (non-treated) are eggplants without Polygonaceae plant strips (n = 3, error bars: SE).

To determine the role of Polygonaceae plants as potential Japanese native insectary plants in conserving and promoting INEs, we performed field studies. Population trends of major pests such as aphids, thrips, and whiteflies were determined together with INEs such as coccinellids, green lacewings, and *Orius* spp. Results shows that, while there was no significant difference between the treatments (eggplants with and without Polygonaceae plant strips) for the aphids (*p* = 0.4675), some marginal differences could be observed when taking into consideration the effect of sample intervals (*p* = 0.0717) and the interaction between treatments and sampling intervals (*p* = 0.0627). On the other hand, whiteflies (*p* = 0.0011) and thrips (*p* = 0.0000) were significantly reduced in eggplants with Polygonaceae plant strips, as with the effects of sampling intervals (*p* = 0.0155 for whiteflies and *p* = 0.0000 for thrips) as well as the effect of the interaction between the treatment and sampling intervals (*p* = 0.0037 for whiteflies and *p* = 0.0000 for thrips) (Figure 2, Table 2). Similarly, INEs such as green lacewing and *Orius* spp. were observed to be significantly higher on eggplants with Polygonaceae plant strips compared to eggplants without Polygonaceae plant strips (*p* = 0.0241, *p* = 0.0241 for green lacewings and *Orius* spp., respectively) (Figure 2, Table 2), suggesting that *Orius* spp. and green lacewings could be involved in suppressing the thrips and whitefly densities on eggplants. Furthermore, to determine if pests, as well as INEs were present on the Polygonaceae plants, we also surveyed the pests and INE trends on the Polygonaceae plant strips. As expected, aphids and thrips (pests) and coccinellids, green lacewings, and *Orius* spp. (INEs) were present and seemed to increase on *P. lapathifolia* as the survey progressed (Figure A2). Whether the higher densities of *Orius* spp. on *P. lapathifolia* migrated to eggplants remains unknown; therefore, we performed laboratory experiments to confirm their species composition and the migration pattern.

### 3.3. Identification and Species Composition of Orius *spp.* on Eggplants and P. lapathifolia

**Figure 3:**
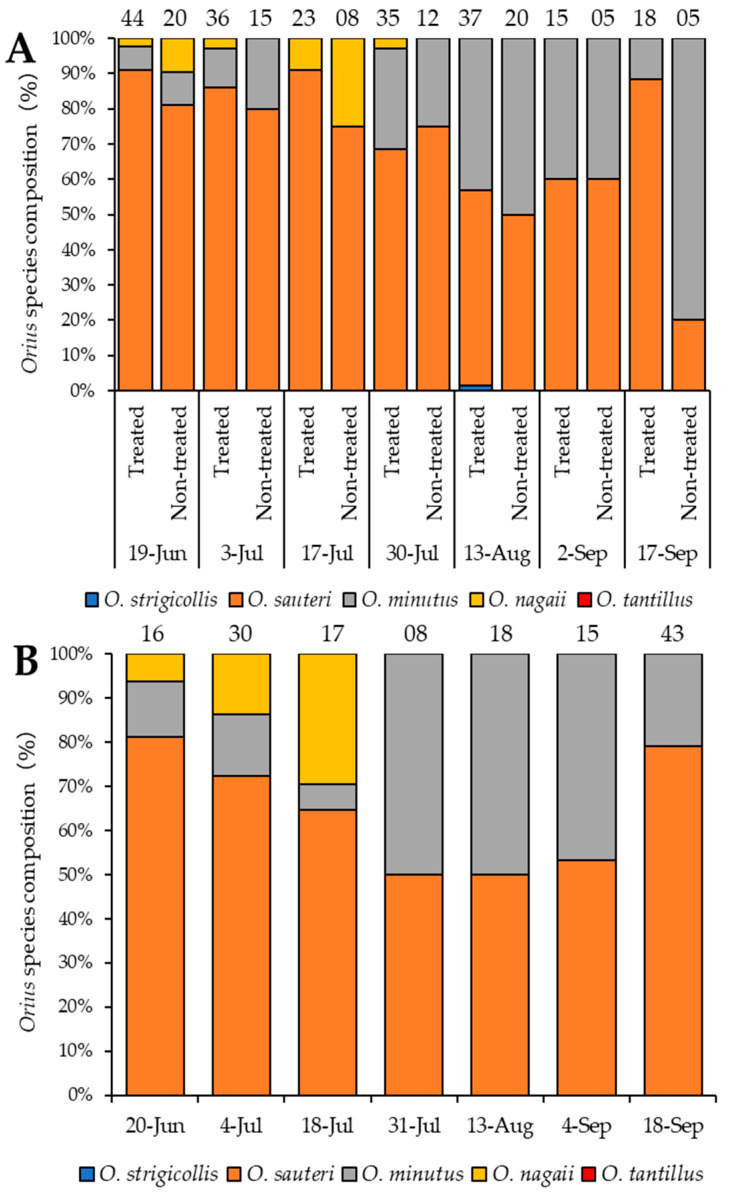
Species composition of *Orius* spp. collected on eggplants (**A**) and on *Persicaria lapathifolia* (**B**). The numbers at the top end of the bar graphs represent the number of *Orius* spp. individuals used for a specified sampling date. *Orius strigicollis* is represented with blue bars, *O. sauteri* with orange bars, *O. minutus* with grey bars, *O. nagaii* with yellow bars, and *O. tantillus* with red bars. Treated and non-treated in ‘**A**’ im-plies eggplants with Polygonaceae plant strips and eggplants without Polygonaceae plant strips, respectively.

Identifying the species that inhabits the given agroecosystem is essentially fundamental in designing a successful CBC. Adult *Orius* spp. individuals sampled during the survey were identified using the multiplex PCR method [17]. There are five known species of *Orius* species distributed in Japan, four on the main island of Honshu, *O. sauteri*, *O. strigicollis*, *O. minutus*, and *O. nagaii* [17], and *O. tantillus* on the Okinawa islands [11]. The species composition of the *Orius* spp. on eggplants and *P. lapathifolia* is shown in Figure 3. *Orius sauteri* seems to be the dominant species at the earlier sampling dates (June and July) on the eggplants (Figure 3A) and on *P. lapathifolia* (late June to mid-July) (Figure 3B). *Orius minutus* sporadically occurred in the earlier dates and seems to increase in its occurrence as the survey progressed. *Orius nagaii* only appeared in surveys between June and July, while *O. strigicollis* was only observed once on eggplants on 13 August. Coincidently, the occurrence pattern of *Orius* spp. on *P. lapathifolia* resembled that of those on eggplants, indicating that *Orius* spp. could be migrating from *P. lapathifolia* to eggplants. Overall, the dominant occurrence of *O. sauteri* on *P. lapathifolia* (and eggplants) in Fukuyama or at least in Hiroshima Pref. in Western Japan suggests that *P. lapathifolia* could be an important host plant species for *O. sauteri*. This information could be critical in designing biological control strategies that involve specific natural enemy and plant species.

### 3.4. Detection of P. lapathifolia DNA in Orius *spp.*

To confirm if the *Orius* spp. (candidate INE used in this set of experiments) migrates from *P. lapathifolia* to eggplants, we designed *P.-lapathifolia*-specific primers and performed PCR to detect *P. lapathifolia* DNA in the gut of adult *Orius* spp. Upon confirming the amplification of *P. lapathifolia* DNA, we performed PCR on *Orius* spp. adult individuals collected from eggplants as well as from *P. lapathifolia*. The results are presented in Table 3 and Table 4. Results show that *P. lapathifolia* DNA was amplified in the gut of adult *Orius* spp. collected from eggplants (Table 3). In an unexpected turn of events, while *P. lapathifolia* DNA was detected in the gut of adult *Orius* spp., collected from eggplants with and without Polygonaceae plant strips, the detection frequency in *Orius* spp. collected from eggplants without Polygonaceae plant strips was higher than that of the eggplants with Polygonaceae plant strips between June and mid-July surveys (Table 3, *p* = 0.03556 for 19 June, *p* = 0.00001 for 3 July, and *p* = 0.00004 for 17 July), suggesting that adult *Orius* spp. could migrate as far as 10 m or beyond in search for prey materials. Adult *Orius* spp. collected on *P. lapathifolia* also confirmed the detection of *P. lapathifolia* DNA in their guts (Table 4). How far laterally the *Orius* spp. can migrate or, rather, for how long before the plant material is completely metabolized and degraded in the gut of the *Orius* spp. remains for further elucidation. Nonetheless, all things considered, although the detection frequencies were low, the fact that *P. lapathifolia* DNA was detected in the gut of an adult *Orius* spp. individual collected on eggplants suggests that *Orius* spp. did migrate from *P. lapathifolia* to eggplants. These results, together with the population trends of INEs and pests on eggplants, suggest that native Japanese Polygonaceae plants can support and promote INEs when interplanted with vegetable crops, resulting in suppression of pest densities.

## 4. Discussion

The introduction of non-native insectary plants to conserve and promote introduced natural enemies seems to be functioning well in managing pest populations. However, the introduced non-native flora and fauna are causing loss of biodiversity and threatening local ecosystems [20,21,22,23]. Native plants that are adapted to the local environmental conditions with minimal risk of becoming invasive are potential alternatives as substitutes to non-native plants. Studies on native plants and/or managing local habitats for conservation of biological control is not a new concept. As native plants vary between geographical locations, so does the plant species used. A range of studies have reported the use of native plants in supporting natural enemies [24,25,26,27,28]. Furthermore, several of these studies have shown that indigenous arthropods have higher tendencies to visit and use native plants to shelter and breed compared to the non-native plants [24,26]. Thus, native plants do have the potential to conserve and promote indigenous arthropod species, especially INEs, which could be significant in CBC in regulating pest populations in vegetable productions.

In Japan, mostly non-native horticultural insectary plants have been used extensively in biological control of pests. As a substitute for these non-native insectary plants, we surveyed the surrounding habitats of organic fields in Western Japan to identify native plants as potential insectary plants. Among the native plants surveyed, we identified Polygonaceae plants as a potential insectary plant. Polygonaceae, especially *P. longiseta* and *P. lapathifolia*, were previously reported to harbor INEs such as phytoseiid mites, important natural enemies of spider mites in fruits (peach) orchards [29]. In North America, *Polygonum* (Polygonaceae) plants are semi-aquatic weeds and are considered occasional pests [30]. There are very few reports demonstrating the role of Polygonaceae plants in promoting and conserving beneficial insects [29,31]. While Polygonaceae plants are mostly referred to as plants of many seeds [32], their somewhat dense flowering could provide floral resources to support arthropod communities, including natural enemies.

Floral resources are essentially vital in supporting diversity of natural enemies, which, in turn, delivers the desired function in pest reduction [33,34,35]. The relatively large number of *Orius* spp. with other INEs observed on *P. longiseta* and *P. lapathifolia* in NARO-WARC fields when planted and managed in a controlled manner suggests that Polygonaceae plants could serve as a useful reservoir, providing refugia, alternative foods, and places to breed, supporting a diversity of arthropod species, including natural enemies and predators. Diversity in natural enemies and predators have, in theory, been linked to delivery of function, i.e., suppression or reduction in pest populations [33,34]. Polygonaceae plants, in part, do support a variety of INEs but does this equate to supporting the delivery of function? Our results, although preliminary, confirmed the movement of adult *Orius* spp. as the model INE between insectary plants and vegetable crop. We verified by detection of *P. lapathifolia* DNA in the gut of an adult *Orius* spp. collected on eggplants, and the almost similar species composition pattern between *Orius* spp. on eggplants and the native plant *P. lapathifolia* suggests that natural enemies do move from insectary plants and vegetable crops. Whether the movement of natural enemies from insectary plants to vegetable crops results in pest reduction, achieving delivery of function, is still debatable as we could only observe significant reduction in two pest species but not all of them. Further studies are needed to comprehensively and conclusively prove that Polygonaceae plants can indeed support diversity of natural enemies, resulting in delivery of function in pest suppression. Nonetheless, we can only infer that Polygonaceae plants may, in part, when integrated with other biological control techniques, support the diversity of natural enemies and the delivery of function in suppression of pests.

Providing an assortment of habitat and resources can support diversity of natural enemies and predators, as a result, enhancing biological control services. This is a known concept that is widely reviewed [36,37,38] and proven in a variety of field experiments [35,39,40,41,42]. Replacing the already known and proven non-native invasive insectary plants is a task that will take time and effort. However, for the sake of preserving the biodiversity and maintaining a healthy ecosystem, native plants are the way forward. Furthermore, to achieve a very effective CBC approach with native plants, one native plant may not be enough. Predictive studies have shown that plant diversity promotes pest reduction through movement patterns, host interaction, and predation from natural enemies [34]. In addition, studies have shown that INE species distribution and abundance can be influenced by the host plant chemistry and features (flower/leaf morphology and architecture), plant origin (native), in addition to the prey and the vegetative sites it provides for mating, breeding, shelter, and hibernation [43,44,45,46,47]. This study with its preliminary results has set the basis that INE species can breed and proliferate on native (flowering) plants. Here, we suggest further studies on searching and screening for potential native insectary plants that can support a diversity of INEs. In this digital age where mundane man-hours in ecological surveys can be approached with revolutionary sequencing technology, i.e., Next-Generation Sequencing (NGS), native plant search and screening using the environmental DNA (eDNA) technology can be a useful approach to identifying potential native insectary plants. Furthermore, to maximize delivery of function in pest suppression, multiple annual field studies and laboratory experiments in tracing natural enemy movements, pest consumption rates, pest–natural enemy–native insectary plant interaction studies are a prerequisite.

## 5. Conclusions

In this era where climate crisis and movement of invasive species between borders are threatening local biodiversity and ecosystems, urgent actions are a prerequisite. In conservation biological pest control, flowering plants are utilized to provide food (pollen and nectar), shelter, and breeding grounds to support natural enemies/predators. However, most of the flowering plants are non-native invasive plants. Ecologically sound yet noninvasive substitutes in the likes of native plants are suggested. To replace the already available non-native invasive insectary plants will be a task too tedious to achieve in the given timeframe. To realize this urgency is already a step forward and progress nonetheless. Our field studies have identified Polygonaceae plants with a plethora of other native plants as potential native plants that can support INEs. Results are still preliminary and, therefore, further experiments are needed to elucidate the role of Polygonaceae plants as well as other native plants in supporting the abundance and diversity of INEs that can yield the desired delivery of function through pest suppression.

## Figures and Tables

**Table 1:** List of native plants surveyed (between 2023 to 2024) from the surrounding habitats of organic fields in Western Japan.

Study Site	Plants Observed		July	August	September	October
	Family	Species				
Fukuyama	Asteraceae	*Cirsium japonicum* DC. (1838)	**■**	▲	▲	■
	Asteraceae	*Eupatorium japonicum* Thunberg ex Murray.	▲●	▲●	▲●	▲●
	Caryophyllaceae	*Dianthus superbus* L.	■	■	■	■
	Caprifoliaceae	*Patrinia scabiosifolia* Link	▲●	▲●	▲●	▲●
	Commelinaceae	*Commelina communis* L.	▲●	▲●	▲●	▲●
	Fabaceae	*Trifolium pratense* L. ^†^	▲●	▲	▲●	□
	Lamiaceae	*Ocimum basilicum* L. ^†^	▲●	▲●	▲●	▲●
	Lamiaceae	*Ocimum tenuiflorum* L. ^†^	□	▲●	▲●	▲●
	Lamiaceae	*Salvia japonica* Thunb.	▲●	□	□	□
	Papaveraceae	*Macleaya cordata* (Willd.) R. Br.	□	□	□	□
	Polygonaceae	*Persicaria longiseta* (Bruijn) Kitagawa 1937	▲●	▲●	▲●	▲●
	Polygonaceae	*Persicaria lapathifolia* (L.) Delarbre 1800	▲●	▲●	▲●	▲●
Higashi-Hiroshima	Acanthaceae	*Rostellularia procumbens* (L.) Nees (1847)	□	□	●	□
	Commelinaceae	*Commelina communis* L.	□	■	▲●	▲
	Onagraceae	*Oenothera biennis* L. *	□	□	▲●	□
	Polygonaceae	*Persicaria longiseta* (Bruijn) Kitagawa 1937	□	▲	▲●	■
	Polygonaceae	*Persicaria pubescens* (Blume) H. Hara	□	□	▲	●
	Polygonaceae	*Polygonum thunbergii* Sieb. & Zucc.	□	□	□	▲
Jinsekikogen-town	Apiaceae	*Cnidium officinale* Makino *	□	□	▲	□
	Apiaceae	*Ostericum sieboldii* (Miq.) Nakai	□	□	□	▲
	Asteraceae	*Aster yomena* (Kitam.) Honda	□	□	□	▲
	Asteraceae	*Eclipta prostrata* (L.) L.	□	▲●	▲●	□
	Asteraceae	*Sigesbeckia pubescens* (Makino) Makino	□	□	□	▲
	Balsaminaceae	*Impatiens textori* Miq.	□	□	▲●	▲●
	Brassicaceae	*Barbarea vulgaris* W.T. Aiton	□	▲	▲	□
	Commelinaceae	*Commelina communis* L.	□	▲●	▲●	▲●
	Fabaceae	*Lotus corniculatus* L.	□	▲	□	□
	Lamiaceae	*Clinopodium multicaule* (Maxim.) O. Kuntze. var. multicaule	□	□	■	□
	Lamiaceae	*Isodon inflexus* (Thunb.) Kudo	□	□	□	▲
	Lamiaceae	*Mosla scabra* (Thunb.) C.Y. Wu et H.W. Li	□	□	□	▲
	Lamiaceae	*Salvia japonica* Thunb.	□	▲●	▲●	□
	Onagraceae	*Circaea mollis* Siebold & Zucc.	□	▲●	□	□
	Rosaceae	*Agrimonia pilosa* Ledab. var. japonica	□	▲	▲	□
	Rubiaceae	*Paederia foetida* L.	□	▲●	□	□
	Polygonaceae	*Persicaria filiformis* (Thunb.) Nakai	□	□	▲	□
	Polygonaceae	*Persicaria longiseta* (Bruijn) Kitagawa 1937	□	▲●	▲●	▲●
	Polygonaceae	*Persicaria orientalis* (L.) Spach *	□	▲●	▲●	▲●
	Polygonaceae	*Polygonum thunbergii* Sieb. & Zucc.	□	□	□	▲

▲: Herbivores observed, ●: natural enemies present, ■: no arthropod (including natural enemies and pests) species observed, □: plants not available during survey, *: non-native but neutralized in Japan, ^†^: non-native insectary plants.

**Table 2:** Results of the partial GLM analysis showing the interaction effects between treatments and sampling intervals on the pests (aphids, whiteflies, and thrips) and INEs (coccinellids, green lacewings, and *Orius* spp.) on eggplants.

Arthropod Species Observed		Response								
		Treatments			Sampling Intervals			Treatment × Sampling Intervals		
		*d.f*	*F*	*p*	*d.f*	*F*	*p*	*d.f*	*F*	*p*
Pests	Aphids	1	0.934	0.4675	8	2.094	0.0717	8	2.170	0.0627
	Whiteflies	1	4.847	0.0011	8	2.964	0.0155	8	3.791	0.0037
	Thrips	1	19.464	0.0000	8	13.049	0.0000	8	14.978	0.0000
INEs	Coccinellids	1	1.321	0.2711	8	1.198	0.3241	8	1.244	0.3015
	Green lacewings	1	2.868	0.0241	8	2.089	0.0723	8	2.288	0.0510
	*Orius* spp.	1	5.406	0.0005	8	3.170	0.0108	8	4.952	0.0010

**Table 3:** Detection frequency of *Persicaria lapathifolia* DNA in the guts of adult *Orius* spp. collected on eggplants.

	19-June		03-July		17-July		30-July		13-August		02-September		17-September	
	Treated *	Non-Treated #	Treated	Non-Treated	Treated	Non-Treated	Treated	Non-Treated	Treated	Non-Treated	Treated	Non-Treated	Treated	Non-Treated
Tot. No. of *Orius* individuals observed	44	20	36	15	23	8	35	12	37	20	15	5	18	5
No. of *Orius* spp. that showed *P. lapathifolia* DNA amplification	3	4	1	4	4	2	0	1	3	0	0	0	1	0
Detection frequency (%)	6.8	20.0	2.8	26.7	17.4	25.0	0.0	8.3	8.1	0.0	0.0	0.0	5.6	0.0
χ2 (*p*-value) at α = 0.05	0.03556		0.00001		0.00004		0.12469		0.35644		1.00000		0.03868	

*: Adult *Orius* spp. collected from the eggplants with Polygonaceae plant strips, #: adult *Orius* spp. collected from the eggplants without Polygonaceae plant strips.

**Table 4:** Detection frequency of *Persicaria lapathifolia* DNA in the guts of adult *Orius* spp. collected on *P. lapathifolia*.

	20-June	04-July	18-July	31-July	13-August	04-September	18-September
Tot. No. of *Orius* individuals observed	16	30	17	8	18	15	43
No. of *Orius* spp. that showed *P. lapathifolia* DNA amplification	3	5	3	0	1	4	4
Detection frequency (%)	18.8	16.7	17.6	0.0	5.6	26.7	9.3

## Data Availability

The data presented in this study are available upon request from the corresponding author.
